# The role of off-equatorial surface temperature anomalies in the 2014 El Niño prediction

**DOI:** 10.1038/srep19677

**Published:** 2016-01-20

**Authors:** Jieshun Zhu, Arun Kumar, Bohua Huang, Magdalena A. Balmaseda, Zeng-Zhen Hu, Lawrence Marx, James L. Kinter III

**Affiliations:** 1 Climate Prediction Center, NOAA/NWS/NCEP, College Park, Maryland, USA; 2 Innovim, Greenbelt, Maryland, USA; 3 Center for Ocean-Land-Atmosphere Studies, George Mason University, Fairfax, Virginia, USA; 4 Department of Atmospheric, Oceanic, and Earth Sciences, College of Science, George Mason University, Fairfax, Virginia, USA; 5 European Centre for Medium-Range Weather Forecasts, Reading, United Kingdom

## Abstract

The 2014 El Niño, anticipated to be a strong event in early 2014, turned out to be fairly weak. In early 2014, the tropical Pacific exhibited persistent negative SST anomalies in the southeastern Pacific and positive SST anomalies in north, following the pattern of the Southern Pacific Meridional Mode. In this study, we explored the role of the off-equatorial SST anomalies in the 2014 prediction. Our experiments show that 40% of the amplitude error at the peak phase could be attributed to the lack of prediction of negative SST anomalies in the southeastern Pacific. However, the impact of this model error is partially compensated by the absence of the positive SST anomalies in the tropical western North Pacific in the model. The model response to the amplitude of negative southeastern Pacific SST anomalies is nonlinear in terms of equatorial warming, because the enhanced meridional pressure gradient forces very strong meridional winds without accelerating the zonal wind component near the equator. Our study suggests that reliable forecasts of ENSO strongly rely on correctly modeling the meridional SST gradient, as well as its delicate feedback with the zonal (ENSO) mode.

Since initial attempts around three decades ago^1^, the ability of dynamical models to predict the El Niño and the Southern Oscillation (ENSO) has improved significantly^2^,^3^,^4^,^5^,^6^. Operational seasonal ENSO predictions are now routine using coupled general circulation models (CGCMs) in many major climate centers worldwide^7^. It has been demonstrated that, on average, the SST variability in the tropical Pacific associated with ENSO can be successfully predicted several seasons ahead, as quantified based on statistical metrics (e.g., anomaly correlation).

Despite advancements in dynamical predictions and improvements in average skill, on occasion ENSO predictions also have notable failures, two notable examples being the false alarms in 2012 and 2014. In the summer of 2012, there were clear precursors suggesting that an El Niño event was developing in the tropical Pacific, a notion that was supported by a majority of seasonal prediction systems that predicted the occurrence of an El Niño peaking in the subsequent winter (http://iri.columbia.edu/our-expertise/climate/forecasts/enso/archive/201208/QuickLook.html). This, however, turned out to be a false alarm. An even more dramatic, and much publicized, event occurred in 2014. Prompted by remarkable similarities of the oceanic and atmospheric states between early 1997 and 2014 (http://www.cpc.ncep.noaa.gov/products/GODAS/ocean_briefing_gif/global_ocean_monitoring_2014_04.pdf), many climate scientists and forecasters predicted that an El Niño of strength similar to the 1997 “El Niño of the century” could develop in 2014^8^. Most climate models also predicted the occurrence of a strong El Niño peaking in the subsequent winter (http://iri.columbia.edu/our-expertise/climate/forecasts/enso/2014-April-quick-look). However, this turned out to be another false alarm.

Studies have been devoted to understanding why these events did not develop as anticipated. Su *et al*.^9^ argued that the abrupt termination of 2012 warming was mainly controlled by the anomalous SST cooling in the northern subtropical Pacific, which decoupled the equatorial SST warming from convection, wind, and oceanic subsurface variability over the equatorial Pacific. For the 2014 case, Menkes *et al*.^10^ attributed the absent warm event to the lack of Westerly Wind Events (WWEs) from April to June, while Chen *et al*.^11^ emphasized the combined effects of WWEs and build-up of equatorial heat content. An observational analysis by Min *et al*.^12^, on the other hand, argued that the 2014 El Niño development may mainly be hindered by the existence of negative SST anomalies in the southeastern Pacific.

In this study, we focus on the prediction of 2014 event. One aspect that differentiates this event from 1997 was the presence of strong SST anomalies in the off-equatorial Pacific in 2014^13^. In particular, there were strong negative SST anomalies in the southeastern Pacific in early 2014 (Fig. 1b,d), which together with the positive SST anomalies in north resemble the SST pattern related to the Southern Pacific Meridional Mode (SPMM)^14^. Based on an operational prediction system^15^, we will explore how the 2014 El Niño prediction is influenced by these off-equatorial SST anomalies, including those over the southeastern Pacific and the tropical western North Pacific as well.

We first evaluate the performance of ENSO prediction in the control experiment (i.e., CTL; see Methods) by comparing with observations. For observations (Fig. 1, black curve), the Niño-3.4 index fluctuated around 0.5 °C during April 2014 – March 2015 (black curve in Fig. 1a), generally representative of a neutral state with a fairly weak warming. In contrast, CFSv2 predicted the occurrence of an El Niño event. For most ensemble members (green curves in Fig. 1a), the predicted Niño-3.4 SST anomalies increased at about a constant rate from April until November and decayed afterwards, with Niño-3.4 SST anomalies ranging from 1.9 °C to 3.3 °C in November. What’s more important, after a gradual increase from −0.5 °C in February to 0.5 °C in June, the observed Niño-3.4 index abruptly decreased from June to July, whereas none of the CTL members captured this evolution. The dramatic difference suggests that *June* might be the vital month for the ENSO evolution in 2014, contributing to the difference between observations and predictions.

In June 2014, outside the equatorial region, there were two regions with contrasting difference between observations and CTL prediction in terms of distribution of SST anomalies (Fig. 1d vs. 1e). In the eastern Pacific south of the equator, the observed anomalies were below −0.5 °C. These anomalies can be traced back to the winter of 2013/2014 (see April in Fig. 1b), and sustained themselves until the late summer (not shown) probably through the wind-evaporation-SST (WES) feedback and the positive stratus-SST feedback^14^, as seen from Fig. 2a,b, respectively. Although the SST anomalies were properly initialized in CFSv2 (Fig. 1c), the negative SST anomalies decay quickly and are not present in June (Fig. 1e), which is a deficiency exhibited by CFSv2 and other prediction systems (e.g., the ECMWF prediction system, not shown). The deficiency confirms that the thermodynamic WES and stratus-SST feedbacks are not well reproduced by current state-of-the-art models^16^,^17^, as seen from Fig. 2c,d, respectively. The second region is the tropical western North Pacific where positive SST anomalies are evident in observations (Fig. 1d) but absent in CTL prediction (Fig. 1e). Compared with the cold SST anomalies in the southeastern Pacific, the observed positive SST anomalies were not well-organized until May/June and also decayed more quickly during the summer (figures not shown). The positive SST anomalies also seem relevant to the thermodynamic WES process (Fig. 2c). They could be part of the above meridional mode that CFSv2 failed to predict, but also could be due to more regional processes, such as the effect of some persistent weather systems. The errors in simulating/predicting the two off-equatorial SST anomalies suggest that not only the local dynamics and physics (e.g., WES and status-SST feedbacks), but also the inter-basin interaction is not well represented in current climate models.

Considering the failed prediction in 2014 and the media attention it generated^8^,^18^, one may ask what would have happened if the off-equatorial temperature anomalies were better predicted. In the following, three sensitivity hindcast experiments are conducted to address this question. In these three experiments, the off-equatorial temperature anomalies in June with different configurations are artificially added to the April ocean initial conditions (see Methods for full details). In particular, in SE the observed cold SST anomaly in the southeastern Pacific (180°W-70°W, 30°S-Eq; the southeastern purple rectangle in Fig. 1d) was added, and the same anomaly with triple the magnitude was added in hindcast SE3. In hindcast SEpTNWP, the same anomaly together with the warm anomaly in the tropical northwestern Pacific (120°E–180°E, 10°S–20°N; the northwestern purple rectangle in Fig. 1d) was added. The experimental design intends to extend the existence of the off-equatorial anomalies until June in CFSv2 predictions.

It should be noted that as is the case with most sensitivity experiments, our experimental setup might have some inconsistencies. This is because the seasonality of anomalies imposed and their influence may not be realistic in the context of the seasonal cycle. For example, the sensitivity experiment to address the role of the positive TNWP SST anomalies, i.e., adding the positive June TNWP SST anomalies to April initial conditions, could overestimate their effect in 2014 El Niño prediction. This is because the positive TNWP SST anomalies were taken from an inactive Madden-Julian Oscillation season (i.e., June) and were implanted in an active season (i.e., April), which may initiate some westerly wind bursts in the western Pacific in addition to bringing some systematic wind changes.

Figure 3 shows the predicted Niño-3.4 indices in three sensitivity hindcasts together with those in CTL and observations. To highlight the effect of the off-equatorial temperature anomalies, Figs 4 and 5 present the ensemble mean difference between the three sensitivity hindcasts and CTL, with the former (latter) demonstrating the difference of SST and surface wind stress anomalies (upper-ocean temperature along the equator) in three hindcasts relative to CTL in April, June and December.

In hindcast SE, an El Niño event developed as in CTL, but the predicted amplitude was clearly lower than that in CTL (Figs 3 and 4a–c). In terms of ensemble mean Niño-3.4 index (Fig. 3), both CTL and SE reached a peak in November 2014, with a magnitude of 2.38 °C and 1.75 °C respectively in contrast to 0.78 °C in OISSTv2, which suggests that around 40% of ensemble mean amplitude errors in CTL could be attributed to the absence of the negative SST anomalies in the southeastern Pacific. The amplitude improvement in SE over CTL occurs at all lead times, and is significant at most lead times at the 95% confidence level (Fig. 3). In fact, if the ensemble spread (i.e., the variance among the 20 ensemble members) is taken into account, the predicted Niño-3.4 indices from CTL and SE were well separated since September 2014.

The improvement in SE over CTL is related to the surface wind differences initiated by the addition of negative SST anomalies in the southeastern Pacific in SE. In particular, as a result of the inserted negative SST anomalies, the Inter Tropical Convergence Zone (ITCZ) convergence located to the north of the equator was enhanced throughout the basin (Fig. 4a,b), driven by the strengthened southeast trade winds that also extend further north from the southern tropical Pacific (Fig. 4a,b). The dynamics of this response are similar to the atmospheric response to the anomalous cross-equatorial SST gradient (or the meridional mode) in the tropical Atlantic^19^,^20^,^21^. As part of the response, there were stronger meridional cross-equatorial flows in the eastern Pacific and stronger easterly winds appear near the equator (Fig. 4a,b). The wind difference is small at the beginning (Fig. 4a), but strengthens and extends equatorward (see Fig. 4b for June) as the positive WES feedback comes into effect. Both the meridional and zonal wind stress response work in the direction of cooling the upper ocean in the eastern Pacific (Fig. 5b).

Even though the negative SST anomalies in the southeastern Pacific were added in the SE experiment, the off-equatorial SST anomalies are still slightly weaker than their observational counterpart by June (figures not shown), reconfirming the model’s deficiency in representing the WES and stratus-SST feedbacks^16^,^17^.

The inserted negative SST anomalies in the initial state are tripled in hindcast SE3. The SST increase substantially amplifies the difference in trade winds in the southern tropical Pacific at the beginning (i.e., April; Fig. 4d vs. 4a). Specifically for the zonal component, in April a contour of −0.01 N/m^2^ is evident on the equatorial side of the inserted SST anomalies in the SE3 difference map (not shown), which, is absent in corresponding map for the SE (not shown). Consistent with the strengthened local easterly wind, stronger subsurface cooling in SE3 than SE also occurs in the eastern Pacific immediately in April (Fig. 5d vs. 5a). Thus, as an immediate consequence of the increase in the negative SST anomalies in the southeastern Pacific, the easterly winds strengthened in the eastern Pacific at the beginning, which could favor a weaker El Niño than SE due to strengthened local Ekman upwelling.

However, the story is different at longer lead times. After the initial strengthening of the easterlies, the strong SST anomalies in SE3 generated so large a meridional gradient that they could trigger very strong meridional winds, without further accelerating the easterlies. This forced the anomalous ITCZ further northward and weakened the trade winds in the northern tropical Pacific. The weakened northeast trade winds in turn warmed the local SST in the northern tropical Pacific as a result of the WES mechanism. This process was evidenced by the cross-equatorial SST and surface wind distributions in June (Fig. 4e), which resembled the meridional mode in the tropical Pacific and Atlantic^14^,^19^,^20^,^21^. Also because of the positive WES feedback, the cross-equatorial pattern develops further after its initiation, as seen from the comparison between June (Fig. 4e) and April (Fig. 4d). The difference between SE and SE3 suggests that the cross-equatorial mode might not be evoked unless the SST anomalies in the southern tropical Pacific are strong enough to exert influence in the northern hemisphere. Due to the stronger merdional orientation of the surface wind anomalies in SE3, the surface winds in June (Fig. 4e vs. 4b) were featured by somewhat weaker easterly anomalies in SE3 than in SE throughout the equator (especially around 120°W), and much stronger meridional winds over the eastern Pacific in SE3. The local meridional and zonal winds in the eastern Pacific in turn exerted opposite effects on ENSO evolutions by modulating the local upwelling, with the former (the latter) favoring a weaker (stronger) El Niño in SE3 than in SE. In addition, the weaker easterly anomalies (i.e., westerly wind difference) in the western and central equatorial Pacific in SE3 (Fig. 4d,e) might also be related to a eastwards propagating warm signal along the equatorial thermocline (Fig. 5d,e) probably by triggering oceanic downwelling Kelvin waves in difference fields. The process, in principle, also favored a stronger El Niño in SE3 than in SE.

Therefore, multiple competing factors seem to influence the responses in SE3 to the amplification of the added negative SST anomalies in the southeastern Pacific. At the beginning, the SST amplification immediately strengthened the easterly winds in the eastern Pacific, as well as a further equatorward extension of the off-equatorial cold SST anomalies, resulting in slightly lower Niño-3.4 index in SE3 than SE during April–May 2014 (Fig. 3). As the forecast proceeds, the WES feedback were in effect for a while, and a cross-equatorial pattern was developed, which is because very strong meridional winds (with a possible reduction in zonal wind stress) were triggered by the large SST meridional gradient in SE3. The compensating effects from merdional and zonal winds result in an essentially negligible SST difference between SE3 and SE in the central and eastern Pacific since June 2014 (Figs 3 and 4b,c,e,f).

When both the positive SST anomalies in the tropical western North Pacific and the southeastern negative SST anomalies were together added in the April ocean initial conditions (OICs), i.e., the SEpTNWP hindcast, the predicted El Niño was stronger than that in SE and SE3, but still weaker than that in CTL (Fig. 3). The amplitude increase over SE/SE3 was related to anomalous equatorial westerly winds over the far western Pacific, which were generated by the positive SST anomalies in the tropical western North Pacific (Fig. 4g). The westerly winds triggered oceanic downwelling Kelvin waves (Fig. 5g). Thus, the role of the southeastern negative SST anomalies was offset by these downwelling Kelvin waves, resulting in a stronger El Niño in SEpTNWP than SE/SE3. These processes were reminiscent of the western Pacific oscillator paradigm for ENSO^22^, in which the off-equatorial SST and sea level pressure variations west of the date line were proposed to initiate equatorial easterly winds over the far western Pacific during an El Niño, contributing to the phase transition to a neutral or La Niña state. However, the scenario here was slightly different (i.e., strengthening rather than weakening the existing eastern Pacific warming), because SST anomalies in the tropical western North Pacific were opposite to those expected from an eastern Pacific warming (Fig. 1c vs. 2 of 23). In addition, the negative SST anomalies in the southeastern Pacific were also visibly weaker in SEpTNWP than in SE, suggesting a weaker WES feedback.

In summary, our experiments suggest that, for the 2014 El Niño prediction, around 40% of amplitude prediction errors at its peak phase (i.e., November or December 2014) could be attributed to lack of the negative SST anomalies in the southeastern Pacific, which was related to model deficiency in reproducing the WES and stratus-SST feedbacks^16^,^17^. The thermodynamical feedbacks, however, are not completely absent in current climate models, but they are generally too weak to sustain the initial SST anomalies in the southeastern Pacific Ocean. Their consequences can be seen in the sensitivity experiments conducted in this study. We suggest that, if the model could predict the Pacific Meridional Mode by representing WES and stratus-SST feedbacks appropriately, forecast errors explained by the SST anomalies in the Southeastern Pacific can be further reduced.

Even though our experiments found a role for the off-equatorial (SE in particular) SST anomalies in the 2014 El Niño prediction, and previous studies also emphasized the role of the Pacific Meridional Mode (see^12^, particularly the contribution of SST anomalies in the southeastern Pacific) in the evolution of ENSO^14^,^24^, our experiments also suggest that the effect of the off-equatorial SST anomalies on the ENSO development is quite subtle and should not be oversimplified. As our experiment SE3 has shown, too large off-equatorial SST anomalies may trigger overly strong WES feedback with large meridional wind anomalies over the equatorial ocean but may not have a strong effect on the zonal wind anomalies. Secondly, in 2014 the effect of SST anomalies in the southeastern Pacific could be offset by the positive SST anomalies in the tropical western North Pacific. In fact, stronger SST anomalies in the southeastern Pacific in SE3 invoked positive SST anomalies in the northern tropical Pacific, which with the original southeastern Pacific SST anomalies played a compensating role in the later stage of the ENSO development. Therefore, a correct simulation of both the off-equatorial SST anomalies and the strength of the WES feedback are important here. Finally, as seen from our experiments for the 2014 case, over 50% forecast amplitude errors could not be explained by the SST anomalies in the southeastern Pacific. In particular, it was not directly responsible for the decay of the Niño3.4 index in June that altered the subsequent evolution of the SST anomaly.

On the other hand, noticeable prediction biases are also seen in the Indian and Atlantic Oceans during the developing phase of the 2014 El Niño event (figures not shown). As previous studies have suggested that the conditions over these basins can influence the ENSO evolutions as well^25^,^26^, it is possible that these biases also played a role in the 2014 El Niño prediction, either affecting the equatorial Pacific directly via atmospheric teleconnection or in an indirect way by first affecting the off-equatorial Pacific (e.g., the study regions in the paper). An attribution statement with any confidence, however, can only be made with additional sensitivity experiments beyond those conducted as part of this study.

## Methods

### NCEP Climate Forecast System, version 2 (CFSv2)

The forecast model used in this study is CFSv2^15^. CFSv2 has been the operational forecast system for seasonal-to-interannual prediction at NCEP since March 2011. In CFSv2, the ocean model is the GFDL MOM version 4, which is configured for the global ocean with a horizontal grid of 0.5° × 0.5° poleward of 30 °S and 30 °N and meridional resolution increasing gradually to 0.25° between 10 °S and 10 °N. The vertical coordinate is geopotential (z-) with 40 levels (27 of them in the upper 400 m), with maximum depth of approximately 4.5 km. The atmospheric model is the global forecast system (GFS), which has horizontal resolution of T126 (105-km grid spacing, a coarser resolution version of the GFS operational weather forecast at NCEP), and 64 vertical levels in a hybrid sigma-pressure coordinate. The oceanic and atmospheric components exchange surface momentum, heat and freshwater fluxes, as well as SST, every 30 minutes. More details about CFSv2 can be found in^15^.

### Hindcast experiments

The ocean initial conditions (OICs) for all hindcast experiments are based on the instantaneous ocean states (restart files) from ECMWF ORAS4^27^. In ORAS4, there are five ensemble members to represent uncertainties in the ocean analysis. Four sets of hindcast experiments are conducted for the 2014 case, all starting from April 2014. For each of them, 20 ensemble members are applied with five ORAS4-OICs paired with four atmosphere/land initial conditions, i.e., the instantaneous states at 00Z of 1^st^–4^th^ April 2014 from the NCEP Climate Forecast System Reanalysis (CFSR^28^).

- In the control experiment (referred as CTL), five OICs are generated from the respective five ORAS4 restart files, without any modifications except the interpolations from the ORAS4 grid to the CFSv2 grid.
- In the SE experiment all settings are the same as CTL, except that the observed cold SST anomalies in the eastern Pacific south of the equator (180°W-70°W, 30°S-Eq) in June 2014 are artificially added in the upper three model ocean levels (i.e., depths of 5 m, 15 m and 25 m) of OICs in April.
- The SE3 hindcast is the same as SE except that the amplitude of the added cold SST anomalies was tripled.
- The SEpTNWP hindcast is to take into account the off-equatorial SST anomaly in the western Pacific. In particular, in addition to the above cold anomalies in the southeastern Pacific in the SE hindcast, the observed warm SST anomalies in the tropical western North Pacific (120 °E–180   °E, 10 °S–20 °N; TNWP) in June 2014 were also added to the OICs of SEpTNWP.

The predicted anomalies for the 2014 case were derived by subtracting a lead time-dependent climatology which was defined based on a set of hindcasts during 1982–2009^6^,^29^,^30^. These hindcasts start from each April of 1982–2009, and extend to the following March (i.e., 12 months). Four ensemble members were applied in the set of hindcasts, based on one out of the above five ORAS4 OICs and four atmosphere/land initial conditions (i.e., the instantaneous fields from 00Z of the first four days in April of each year in CFSR). The set of hindcasts have demonstrated good skill in predicting SST evolutions in the tropical Pacific^6^.

### Verifying data

The predicted monthly mean SST anomalies are validated against the optimum interpolation SST analysis, version 2 (OISSTv2^31^). The surface latent heat flux and low-cloud cover anomalies are validated against the CFSR^28^ data. All analyses and figures are based on monthly mean data.

## Additional Information

**How to cite this article**: Zhu, J. *et al*. The role of off-equatorial surface temperature anomalies in the 2014 El Niño prediction. *Sci. Rep.* **6**, 19677; doi: 10.1038/srep19677 (2016).

## Figures and Tables

**Figure 1:**
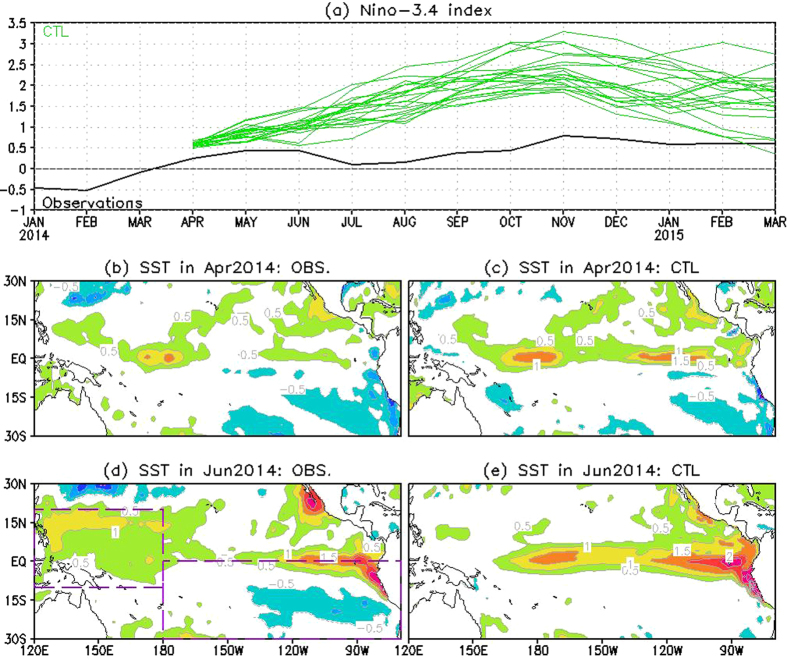
(**a**) Observed (black curve) and CTL predicted (twenty green curves) Niño-3.4 SST anomalies during January 2014–March 2015. (**b,d**) Observed and (**c,e**) CTL predicted ensemble mean SST anomalies over the tropical Pacific in April 2014 (**b,c**) and June 2014 (**d,e**). Contour interval in (**b–e**) is 0.5 °C. Two purple rectangles in (**d**) were used for three sensitivity hindcasts. Figure is generated by Grid Analysis and Display System (GrADS; http://iges.org/grads/).

**Figure 2:**
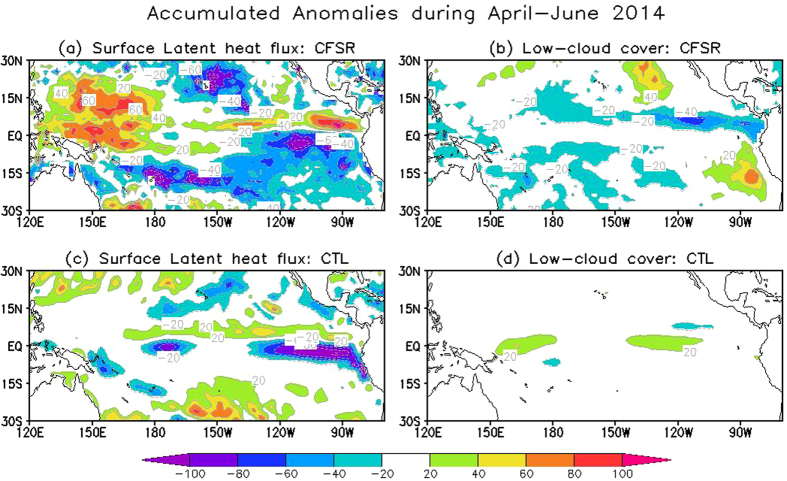
Accumulated anomalies during April–June 2014 over the tropical Pacific for (a,c) surface latent heat flux (W/m^2^) and (**b,d**) low cloud cover (%).(**a,b**) for CFSR (a proxy of observations) and (**c,d**) for CTL predicted ensemble mean. Figure is generated by GrADS.

**Figure 3:**
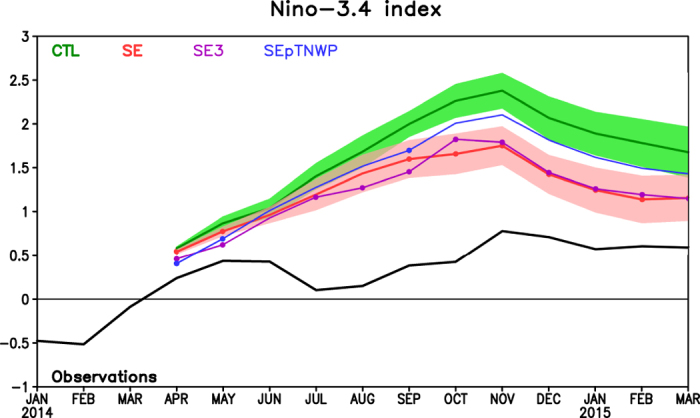
Niño-3.4 SST anomalies (°C) during January 2014–March 2015. Black, green, red, purple and blue curves are for SST anomalies in observations, CTL, SE, SE3 and SEpTNWP, respectively. For forecasts, solid curves represent the ensemble mean. Shaded areas in light green and light red represent ensemble spread for CTL and SE. The dots in red, purple and blue curves means that the ensemble mean SST anomalies in SE, SE3 and SEpTNWP are significantly different from those in CTL at the 95% confidence level. Figure is generated by GrADS.

**Figure 4:**
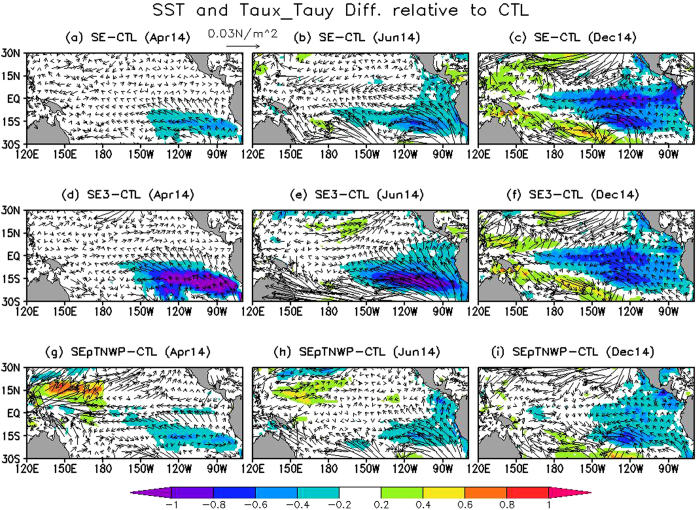
The difference of ensemble mean SST anomalies (shading, °C) and surface wind stress anomalies (vector) in (a–c) SE, (**d–f**) SE3 and (**g–i**) SEpTNWP relative to CTL. (**a,d,g**) for April 2014, (**b,e,h**) for June 2014, and (**c,f,i**) for December 2014. Figure is generated by GrADS.

**Figure 5:**
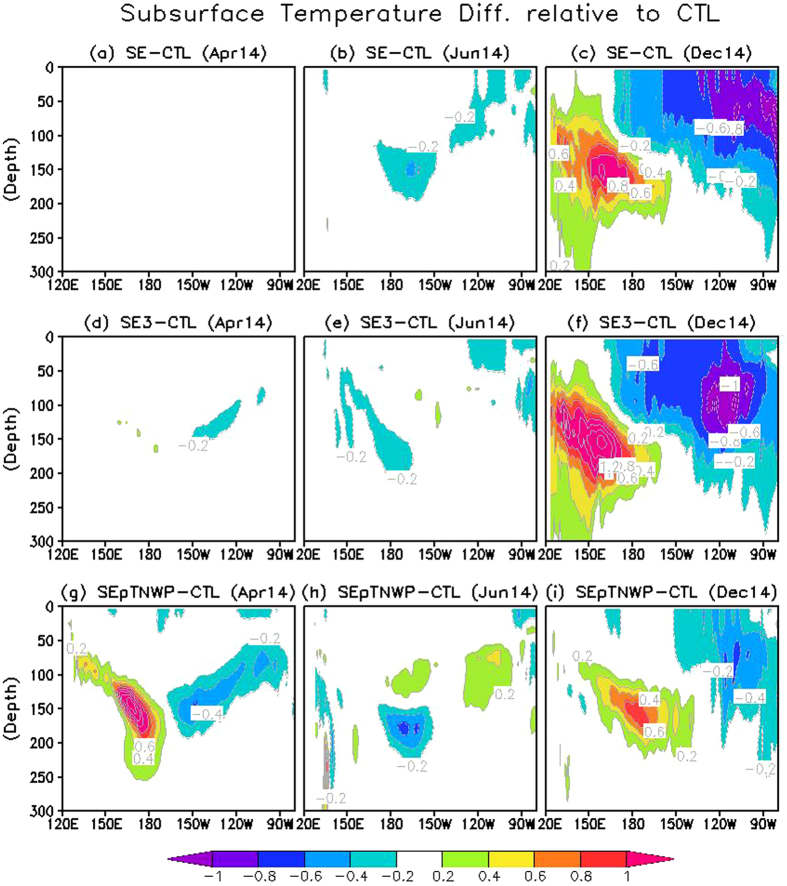
The difference of ensemble mean ocean temperature anomalies (°C) along the equator in (a–c) (SE, (**d–f**) SE3 and (**g–i**) SEpTNWP relative to CTL. **a,d,g**) for April 2014, (**b,e,h**) for June 2014, and (**c,f,i**) for December 2014. Figure is generated by GrADS.
